# Shedding of infectious SARS-CoV-2 by hospitalized COVID-19 patients in relation to serum antibody responses

**DOI:** 10.1186/s12879-021-06202-8

**Published:** 2021-05-27

**Authors:** Hedvig Glans, Sara Gredmark-Russ, Mikaela Olausson, Sara Falck-Jones, Renata Varnaite, Wanda Christ, Kimia T. Maleki, Maria Lind Karlberg, Sandra Broddesson, Ryan Falck-Jones, Max Bell, Niclas Johansson, Anna Färnert, Anna Smed-Sörensen, Jonas Klingström, Andreas Bråve

**Affiliations:** 1 Department of Infectious Diseases, Karolinska University Hospital, SE-141 86 Stockholm, Sweden; 2 Department of Medicine Solna, Karolinska Institutet, Stockholm, Sweden; 3 Center for Infectious Medicine, Department of Medicine Huddinge, Karolinska Institutet, Stockholm, Sweden; 4 Department of Microbiology, Public Health Agency of Sweden, Solna, Sweden; 5 Division of Immunology and Allergy, Department of Medicine Solna, Karolinska Institutet, Stockholm, Sweden; 6 Karolinska University Laboratory, Division of Transfuision Medicine and Clinical Immunology, Karolinska University Hospital, Stockholm, Sweden; 7 Department of Microbiology, Tumor and Cell Biology, Karolinska Insitutet, Stockholm, Sweden; 8 Department of Perioperative Medicine and Intensive Care, Karolinska University Hospital, Stockholm, Sweden; 9 Department of Physiology and Pharmacology, Karolinska Institutet, Stockholm, Sweden

**Keywords:** COVID-19, SARS-CoV-2, Viral shedding, Culture, Antibodies

## Abstract

**Background:**

Coronavirus disease 2019 (COVID-19), caused by severe acute respiratory syndrome coronavirus 2 (SARS-CoV-2), is a global pandemic. The understanding of the transmission and the duration of viral shedding in SARS-CoV-2 infection is still limited.

**Objectives:**

To assess the timeframe and potential risk of SARS-CoV-2 transmission from hospitalized COVID-19 patients in relation to antibody response.

**Method:**

We performed a cross-sectional study of 36 COVID-19 patients hospitalized at Karolinska University Hospital. Patients with more than 8 days of symptom duration were sampled from airways, for PCR analysis of SARS-CoV-2 RNA and in vitro culture of replicating virus. Serum SARS-CoV-2-specific immunoglobulin G (IgG) and neutralizing antibodies titers were assessed by immunofluorescence assay (IFA) and microneutralization assay.

**Results:**

SARS-CoV-2 RNA was detected in airway samples in 23 patients (symptom duration median 15 days, range 9–53 days), whereas 13 patients were SARS-CoV-2 RNA negative (symptom duration median 21 days, range 10–37 days). Replicating virus was detected in samples from 4 patients at 9–16 days. All but two patients had detectable levels of SARS-CoV-2-specific IgG in serum, and SARS-CoV-2 neutralizing antibodies were detected in 33 out of 36 patients. Total SARS-CoV-2-specific IgG titers and neutralizing antibody titers were positively correlated. High levels of both total IgG and neutralizing antibody titers were observed in patients sampled later after symptom onset and in patients where replicating virus could not be detected.

**Conclusions:**

Our data suggest that the presence of SARS-Cov-2 specific antibodies in serum may indicate a lower risk of shedding infectious SARS-CoV-2 by hospitalized COVID-19 patients.

## Background

By the end of December 2019, a cluster of severe pneumonia cases of unknown origin were reported from Wuhan, Hubei province, China [1]. A novel beta-coronavirus, severe acute respiratory syndrome coronavirus 2 (SARS-CoV-2), was identified and isolated on 7 January 2020 [2], causing the coronavirus disease 2019 (COVID-19) [3]. Today, COVID-19 is a pandemic with significant impact on morbidity and mortality around the world [4].

A better understanding of the transmission, the duration of viral shedding and the dynamics of serological responses is of importance to limit the spread of SARS-CoV-2. Detection of viral ribonucleic acid (RNA) in patients may indicate a risk for spread of the virus, and can be used as a marker to guide recommendations of isolation of potentially infectious patients. SARS-CoV-2-RNA has been detected in the airways of COVID-19-patients over the course of disease, normally up to 12–20 days, but in severe cases for longer periods of time [5–8]. Moreover, SARS-CoV-2 RNA has been detected up to months after symptom onset, and even after symptom resolution [5, 6, 9–15]. Importantly, detection of SARS-CoV-2 RNA by RT-PCR does not necessary mean shedding of infectious virus [16]. Given the high pressure on the health care system during the pandemic, it is important to establish to what extent hospitalized patients are contagious and how long they need to be isolated. Thus, it is essential to improve our understanding of factors important for potential SARS-CoV-2 viral transmission from patients.

Thus far, only a limited number of reports on shedding of infectious viruses from the airways in hospitalized COVID-19 patients have been published. In individuals with mild COVID-19, viable virus has been isolated up to 8 days [9]. Virus could not be isolated after 8 days in mild cases of COVID-19 despite high viral loads [9], nor from samples with less than 10^6^ viral RNA copies/mL [17]. Morever, the timepoint at which virus no longer could be isolated coincided with the detection of antibodies to SARS-CoV-2 in serum [18]. In general, antibody responses are detected in < 40% of the patients within the first week of symptoms, and by day 15 after onset of symptoms, a vast majority has seroconverted [19]. In a study with 253 patients, ranging from asymptomatic cases to severe cases at the intensive care unit, isolation of replicating SARS-CoV-2 from airway samples succeeded in 6% of the samples drawn at day 10 [20].

## Methods

### Aim

In order to better understand the viral transmissibility and the time needed to isolate hospitalized COVID-19 patients, we investigated if infectious SARS-CoV-2 could be cultured from nasopharyngeal and sputum samples in hospitalized COVID-19 patients later than 8 days after onset of symptoms. Futher, we investigated if the absence of SARS-CoV-2-specific antibody responses in serum could predict shedding of infectious virus.

### Sample collection

Patients admitted to the Department of Infectious Diseases or the Intensive Care Unit at Karolinska University Hospital, Stockholm, Sweden, with a symptom duration of > 8 days and with a previous SARS-CoV-2 RNA positive nasopharynx or pharynx sample, were included in the study. One patient, sampled 5 days after onset of symptoms, was included as a positive control for viral shedding.

Nasopharyngeal swabs (NPS), sputum as well as serum samples, were collected from all patients at one time point per patient during May 2020. Samples were stored at + 4 °C until transported to the laboratory where virus culture was initiated within 8 h after sampling. All NPS and sputum samples were analyzed by virus culturing and RT-PCR. Serum samples were analyzed by RT-PCR and for binding and neutralizing antibodies.

### Ethical approval and consent to participate

The study was approved by the Swedish Ethical Review Authority, and all relevant regulations for work with human participants were complied with. Patient samples were obtained according to the Declaration of Helsinki. Patients included in the study provided written informed consent. For sedated patients, the denoted primary contact was asked to provide informed consent for the patients and if applicable subsequently informed consent by the patient was obtained.

### RT-PCR

SARS-CoV-2 RT-PCR were performed on all collected patient samples (NPS, sputum and serum) as well as on supernatants from viral culture. 600 μl Trizol was added to each sample and RNA was subsequently extracted using PSS magLEAD 12gC. Following extraction, RT-PCR targeting the envelope(E)- and RNA dependent RNA polymerase (RdRp)-genes were used to detect the presence of SARS-CoV-2 RNA [21, 22]. A cycle threshold (CT) value above 40 was considered negative using a threshold of 0.1. Viral levels were not further quantified.

### Isolation of live SARS-CoV-2 from patient material

Virus isolation was carried out at a biosafety level 3 (BSL3) laboratory. For the isolation of viable SARS-CoV-2 from patient samples, 100 μL NPS or 100 μL sputum were diluted with 100 μL Dulbecco’s Modified Eagle’s Medium (DMEM) and subsequently inoculated on Vero E6 (ATCC CRL 1586) cells in duplicate. Inoculation was carried out for 1.5 h at 37 °C and 5% CO2, then medium DMEM supplemented with 5% fetal bovine serum (FBS), 10X Antibiotic-Antimycotic, 0.6 μg/mL penicillin, 60 μg/mL streptomycin, 2 mM L-glutamine, 20 mM HEPES) were added. Cells were continuously monitored for cytopathic effect (CPE). After 10 days, the cultures were harvested and supernatants were analyzed with RT-PCR specific for the SARS-CoV-2 E-gene and RdRp-gene [21, 22]. Positive CPE, in parallel with increased levels of virus in the culture as analyzed by RT-PCR, was used to confirm the presence of live virus in the patient samples.

### Sequencing of SARS-CoV-2

The primary clinical samples and samples from the 4 individuals with positive virus cultures were sequenced in order to confirm that the cultured virus originated from the primary sample. Sequencing libraries were constructed using the Ion AmpliSeq SARS-CoV-2 Research Panel (Thermo Fisher Scientific, MA, USA). cDNA was synthesized from SARS-CoV-2 RNA using SuperScript IV VILO Master Mix (Thermo Fisher Scientific, MA, USA) and incubated at 25 °C for 10 min, 50 °C for 10 min and 85 °C for 5 min. Automated library preparation and amplification was performed on an Ion Chef instrument with AmpliSeq Chef reagents and specific SARS-CoV-2 primers (Thermo Fisher Scientific, MA, USA). Libraries were quantified as previously published [23]. Libraries were normalized to a final concentration of 35 pM and prepared for sequencing with the Ion 540 Kit-Chef (Thermo Fisher Scientific, MA, USA) for 200 base-pair reads. Sequencing was performed on an Ion GeneStudio S5 Sequencing System (Thermo Fisher Scientific, MA, USA). Raw reads were trimmed and filtered using default parameters, then analyzed with the Ion Torrent Suite software plugin coverageAnalysis, where an average read coverage of 20x for every amplicon of a sample was required to pass quality control. Variant calling against the SARS-CoV-2 reference sequence (NC_045512.2) was performed by the Ion Torrent Suite software plugin variantCaller.

### Immunofluorescence assay (IFA)

IFA was performed as previously described [22]. Briefly, SARS-CoV-2-infected Vero cells mixed with uninfected Vero cells were used as target cells. Serum samples were heat-inactivated at 56 °C for 30 min prior to analysis. Serum samples were two-fold serially diluted starting at 1:20. The titer of IgG in each serum sample was determined by the inverted dilution factor value for the highest dilution with positive staining with AF488-conjugated AffiniPure Goat Anti-Human IgG Ab (Jackson ImmunoResearch Laboratories).

### Micro-neutralization assay

SARS-CoV-2 neutralization assay was performed as previously described [22]. Heat inactivated (56 °C for 30 min) serum were diluted in a two-fold dilution serie starting from 1:10. Each sample was prepared in duplicates. Each dilution was mixed with an equal volume of 4000 TCID50/ml SARS-CoV-2. After incubation, 100 μL of the mixtures were added to Vero E6 cells on 96-well plates and incubated at 37 °C 5% CO2. Four days later the cells were inspected for signs of CPE by optical microscopy. Each well was scored as either ‘neutralizing’ if less than 50% of the cell layer showed signs of CPE, or ‘non-neutralizing’ if ≥50% CPE was observed.

### Statistics

Statistical analyses were performed using GraphPad Prism software 7.0 for MacOSX (GraphPad Software). Correlation analyses were performed using Spearman’s correlation test.

## Results

### Clinical characteristics

Thirty-six hospitalized COVID-19 patients were enrolled in this study. The majority of the patients were men (*n* = 25, 69%), and the median age was 60.5 years (range 25–77 years) (Table 1). Most patients had an underlying risk factor for severe COVID-19 [24]; cardiovascular diseases, respiratory diseases and immunosuppression (Table 1). Thirty-three (92%) patients received supplemental oxygen treatment sometime during their hospitalization. Fourteen (39%) patients were treated in the intensive care unit, out of which 9 were treated with mechanical ventilation. Fourteen patients received immunomodulatory treatment, and two received remdesivir prior to inclusion in the study (Table 1). Patient samples were collected at a median of 18 days (range 9–53 days) after self-reported onset of symptoms, and at a median of 8 days (range 1–44 days) after positive SARS-CoV-2 diagnostic PCR test (Table 1).

**Table 1:** COVID-19 patient characteristics (*n* = 36)

Cohort characteristics	no.	%
Male	25	69
Age, years, median (range)	60.5 (25–77)	
Symptom onset to sampling, days, median (range)	18 (9–53)	
Days since diagnosis (positive SARS-CoV-2 PCR), median (range)	8 (1–44)	
**Comorbidities**		
Diabetes mellitus type II	13	36
Hypertension	11	31
Lung disease	8	22
Cardiovascular disease	7	19
BMI > 25	6	17
Malignancy	3	8
**Treatment**		
Supplemental oxygen	33	92
Mechanical ventilation	9	25
ICU addmission^a^	14	39
Immunomodulatory drugs^b^	14	39
Antiviral treatment (remdesivir)	2	6

^a^ICU addmission before study sampling (*n* = 11), ICU treatment during study sampling (*n* = 2), ICU treatment after study sampling (*n* = 1)

^b^Treatment before sampling corticosteroids (*n* = 13), tociluzimab (*n* = 2) or anakinra (*n* = 1). Two patients had prednisolone treatment before COVID-19 and had extra corticosteroids added as treatment

### Detection of SARS-CoV-2 RNA in hospitalized COVID-19 patients

Including all nasopharyngeal and sputum samples analyzed 23 (64%) of the 36 patients were positive in SARS-CoV-2 RT-PCR. Fourteen of the nasopharynx samples and 18 of the sputum samples were positive (Table 2).

**Table 2:** Detection of SARS-CoV-2 and specific antibody levels in all COVID-19 patients (*n* = 36)

ID	SARS-CoV-2 PCR (Ct value)			SARS-CoV-2 culture		Anti-SARS-CoV-2 IgG titers	Neutralizing antibody titers	Days after onset of symptom
	NPH	Sputum	serum	NPH	Sputum			
1	19	21	neg	pos	pos	< 20	< 10	16
2	24	28	neg	pos	neg	< 20	< 10	9
3^a^	25	19	neg	pos	neg	40	< 10	9
4^a^	26	20	neg	pos	neg	40	10	11
5	32	24	neg	neg	neg	40	10	9
6	neg	23	neg	neg	neg	40	10	10
7^d^	33	n/a	neg	neg	n/a	320	10	9
8^b^	28	37	38	neg	neg	160	15	10
9	neg	n/a	neg	neg	n/a	320	30	11
10	neg	neg	neg	neg	neg	640	30	12
11	neg	n/a	neg	neg	n/a	40	40	10
12^a^	33	n/a	neg	neg	n/a	1280	40	11
13	34	32	neg	neg	neg^f^	1280	40	20
14^a^	28	n/a	neg	neg	neg	640	60	14
15	neg	31	neg	neg	neg	640	60	18
16	35	neg	neg	neg	neg	1280	60	15
17	35	neg	36	neg	neg	1280	60	48
18^a^	30	29	neg	neg	neg	2560	60	28
19^a^	neg	neg	neg	neg	neg^e^	2560	60	30
20	neg	36	neg	neg	neg	640	80	53
21	neg	34	neg	neg	neg	1280	80	43
22^c^	neg	neg	38	neg	neg	2560	80	18
23^a,d^	26	20	neg	neg	neg	320	120	9
24	neg	36	neg	neg	neg	2560	120	39
25	neg	neg	neg	neg	neg	2560	120	18
26^a^	neg	neg	neg	neg	neg	2560	120	21
27^a^	neg	neg	neg	neg	neg	1280	160	21
28	neg	27	neg	neg	neg	1280	160	43
29^a^	neg	neg	neg	neg	n/a	1280	240	21
30	neg	28	neg	neg	neg	1280	240	14
31	neg	28	neg	neg	neg^e^	1280	240	27
32	neg	neg	neg	neg	neg	2560	240	20
33	neg	neg	neg	neg	neg	2560	240	37
34^a,b^	neg	n/a	neg	neg	n/a	1280	320	22
35	neg	32	neg	neg	neg	2560	320	19
36^a^	neg	neg	neg	neg	neg	1280	560	26

*Abbreviation*: *neg* negative, *NPH* nasopharynx, *pos* positive

^a^treatment with corticosteroids, ^b^treatment with tociluzimab, ^c^treatment with anakinra, ^d^remdesivir treatment

^e^Positive for herpes simplex 1 in PCR

^f^Postive CPE in culture, negative for SARS-CoV-2, herpes simples 1 and 2, cytomegalovirus, adenovirus, bocavirus, enterovirus, metapneumovirus, parainfluenzavirus and rhinovirus

The median time of sampling for the PCR-positive patient group was 16 days (range 10–54 days) after symptom onset and 8 days (range 1–44 days) after positive diagnostic sample (Table 2). Thirteen of the 36 patients were SARS-CoV-2 PCR-negative in both nasopharynx and sputum, they were sampled at a median of 22 days (range 11–38 days) after symptom onset and a median of 12 days (range 2–27 days) after positive diagnostic sample (Table 2).

**Fig. 1:**
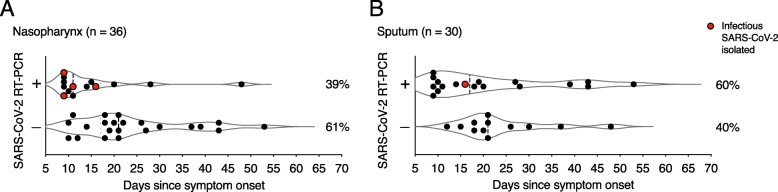
Distribution of positive and negative SARS-CoV-2 PCR samples over time after symptom onset. **a** Distribution of positive (upper row) and negative (lower row) SARS-CoV-2 PCR samples from nasopharynx. **b** Distribution of positive (upper row) and negative (lower row) SARS-CoV-2 PCR sputum samples. Red symbols indicate patients with nasopharynx/sputum samples from which infectious SARS-CoV-2 could be isolated

SARS-CoV-2 RNA was detected by PCR in 39% of the nasopharynx samples (Fig. 1a), and in 60% of the sputum samples (Fig. 1b). SARS-CoV-2 RNA was detected for longer time after onset of symptom in the sputum samples as compared to the nasopharynx samples (Fig. 1). SARS-CoV-2 was isolated in culture from four patients, representing 11% of all patients, and 17% of the SARS-CoV-2 PCR-positive patients (Table 2, Fig. 1). The CT-values in the SARS-CoV-2 PCR for the samples from the culture positive patients ranged between 19 and 26 (Table 2), the CT-values for the sampels from culture negative, but SARS-CoV-2 PCR positive patients ranged between 20 and 35 (Table 2). The primary samples as well as the culture samples from the 4 culture positive patients were sequenced. None of the viral samples showed any significant sequence deviation from the virus variants circulating in the country at that time (data not shown).

All four patients shedding infectious SARS-CoV-2 had underlying diseases, potentially increasing the risk of severe COVID-19 in accordance with [24]; such as underlying lung disease, cardiovascular diseases (hypertension), kidney failure, diabetes mellitus type II, BMI > 25 and hematological malignancy. Three out of the culture positive patients (ID 2, 3 and 4) had been symptomatic for 9–11 days before sampling, and 2 of these patients received betamethasome less than 24 h before sampling. The fourth patient (ID 1), still had viable SARS-CoV-2 in both nasopharynx and sputa on day 16 after onset of symptoms. This patient was immunosuppressed due to a haematological malignancy with leukopenia during hospitalization and had received no COVID-19 treatment, except oxygen and anticoagulants. Three (8%) patients (ID 8, 17 and 22) had detectable but low levels of SARS-CoV-2 RNA in blood, however viable SARS-CoV-2 could not be isolated from respiratory samples of these patients (Table 2).

### SARS-CoV-2-specific antibody responses in hospitalized COVID-19 patients

All but two patients (94%) had detectable levels of SARS-CoV-2-specific immunoglobulin G (IgG) in serum, with IFA-titers ranging from 1:40 to 1:2560. Neutralizing antibodies towards SARS-CoV-2 were detected in 33 of the 36 patients (92%). The three patients without neutralizing antibodies towards SARS-CoV-2 all secreted viable SARS-CoV-2 and the fourth patient secreting viable virus had a low neutralizing titer (1:10) (Table 2). As expected, the positive control patient, sampled at day 5 after onset of symptoms, was positive for infectious virus in the airways and had no detectable SARS-CoV-2-specific antibodies (data not shown).

**Fig. 2:**
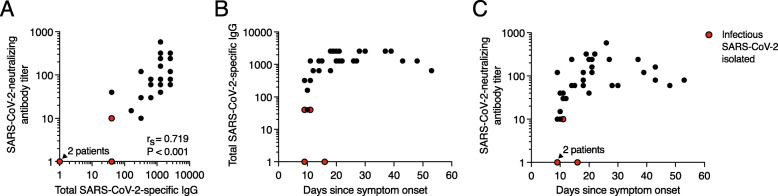
SARS-CoV-2-specific antibody responses **a**. Correlation between anti-SARS-CoV-2-specific IgG titers and neutralizing antibody titers. **b** SARS-CoV-2-specific antibody titers over time since symptom onset. **c** SARS-CoV-2-neutralizing antibody titers over time since symptom onset. Red symbols indicate patients with nasopharynx/sputum samples from which infectious SARS-CoV-2 could be isolated

We observed a strong positive correlation between total SARS-CoV-2-specific IgG titers and neutralizing antibody titers (Spearman’s correlation: rs = 0.719, *p* < 0.001) (Fig. 2a). Patients with SARS-CoV-2 IgG titers equal to or below 1:40 either displayed no detectable, or low levels of neutralizing antibodies (Table 3, Fig. 1a). We also observed generally higher levels of SARS-CoV-2 IgG titers and neutralizing antibody titers in patients sampled late after symptom onset (Fig. 2b-c).

**Table 3:** SARS-CoV-2-specific antibody titers in relation to detectable SARS-CoV-2 RNA and infectious SARS-CoV-2 in nasopharynx in all COVID-19 patients (*n* = 36).

**Total SARS- CoV-2 IgG titer**	**Number of patients**	**PCR Positive**		**PCR negative**		**Culture positive**		**Culture negative**	
		**no.**	**%**	**no.**	**%**	**no.**	**%**	**no.**	**%**
< 20	2	2	100	0	0	2	100	0	0
20 to 40	5	3	60	2	40	2	40	3	60
160 to 320	4	3	75	1	25	0	0	4	100
640 to 2560	25	6	24	19	76	0	0	25	100
**Neutralizing antibody titer**	**Number of patients**	**PCR Positive**		**PCR negative**		**Culture positive**		**Culture negative**	
		**no.**	**%**	**n**	**%**	**no.**	**%**	**no.**	**%**
< 10	3	3	100	0	0	3	100	0	0
10 to 15	5	4	67	2	33	1^a^	17	5	83
30 to 120	18	7	41	10	59	0	0	17	100
160 to 580	10	0	0	10	100	0	0	10	100

^a^neutralizing titer 10

## Discussion

Despite the ongoing SARS-CoV-2 pandemic, the understanding of the dynamics of viral shedding and antibody responses is still limited. We conducted a cross-sectional study on 36 hospitalized COVID-19 patients to assess potential contagiousness of the patients in a hospital setting during May 2020. We were able to isolate viable SARS-CoV-2 from four out of 23 PCR-positive patients. Three of the four patients with infectious/viable SARS-CoV-2 in the airways were sampled within 11 days after symptom onset. However, the fourth patient, who was immunocompromised at the time of inclusion, was sampled at day 16 after symptom onset, indicating that infectious/viable SARS-CoV-2 virus can be shed rather late during COVID-19 in immunocomprised patients. We were not able to detect viable SARS-CoV-2 virus from patients displaying SARS-CoV-2-specific IgG titers above 40, or neutralizing antibody titers above 10. Seroconversion, with detectable neutralizing antibodies, was observed in all 32 patients from whom no viable SARS-CoV-2 virus could be isolated from the respiratory tract. In addition, as expected, no infectious virus could be isolated from the patients that were SARS-CoV-2 RNA PCR negative. There was a large variation in total specific IgG and neutralizing antibody titers in patients sampled early after symptom onset, while patients sampled late after symptom onset (i.e. more than 20 days), showed similar antibody levels.

Our data suggest that individuals with low, or undetectable, levels of SARS-CoV-2 antibodies have an increased risk of shedding infectious virus. This is in concordance with a recent report, with 129 severely ill COVID-19 patients, where infectious virus was found for up to 20 days, median 8 days, after symptom onset [25]. Determinants for the detection of infectious SARS-CoV-2 in that study were either a viral load above 7 log10 RNA copies/mL in airways or a neutralizing antibody titer below 20 in serum [25]. Prior reports of severe acute respiratory syndrome coronavirus 1 (SARS-CoV-1) infection also show similar data, where SARS-CoV-1 could be isolated during the first 2 weeks, but not after 22 days of illness [26]. In the context of SARS-CoV-2, multiple case reports with contradicting data have been published. For example, in one case report a patient was shedding infectious SARS-CoV-2 virus 18 days after symptom onset, despite seroconversion at day 10 and symptom resolution at day 9 [10]. To better understand the correlation between viral shedding and antibody response in COVID-19 patients, large studies need to be performed with focus on the isolation of virus and not solely viral RNA detection. Additionally, case reports on re-infection of SARS-CoV-2 have been reported since the beginning of the pandemic [27], and reinfections with SARS-CoV-2 have been reported both from individuals with, as well as without, antibodies from previous SARS-CoV-2 infection [28–31]. In our study, we only show the development of neutralizing antibodies in the context of viral shedding in SARS-CoV-2 naïve patients. Thus, our results cannot be applied to patients with SARS-CoV-2 reinfection.

The World Health Organization (WHO) recommends at least 10 days after symptom onset plus an additional 3 days without fever and respiratory symptoms, before releasing COVID-19 patients from isolation regardless of isolation location or disease severity [32]. Except for in one immunocompromised patient with 16 days of symptoms, we could not detect infectious/viable SARS-CoV-2 later than 11 days of symptoms. This finding is in concordance with other studies detecting viable viruses long after symptom onset in immunocompromised patients [10, 11, 15, 33], where live virus has been detected for up to 61 days in patients with recently immunosuppression due to a hematological malignancy [34]. In the latter study patients with live virus > 20 days remained seronegative for antibodies to viral nucleoprotein, similar to our findings [34]. As our study took place during May 2020, which was prior to the emergence of the more recent virus variants of concern (e.g. B.1.1.7, P.1, B.1.351) [35–37], additional studies on shedding in patients infected with other variants of SARS-CoV-2 would be of interest.

We confirm a previous report [22] of a strong correlation between total SARS-CoV-2-specific IgG titers and neutralizing antibody titers. Our data also suggest that viable virus may only be present in airways from patients with low or undetectable SARS-CoV-2 antibody titers. Moreover, in the context of immunosuppressed patients cultures from respiratory samples might be necessary in conjunction with serology assays for SARS-CoV-2 antibodies to release the patient from isolation in a hospital setting, although conducting large scale culturing of virus from clinical samples is a challenge as it requires BSL3 laboratory as well as it is time and resource consuming.

## Conclusion

Our data suggests that serological assays may help in providing guidance in judging if a RT-PCR patient may be shedding infectious SARS-CoV-2 virus or not, also in the case of immunocomprised COVID-19 patients.

## Data Availability

The datasets used and/or analysed during the current study are available from the corresponding author on reasonable request.

## Abbrevations

BMI: Body mass index
COVID-19: Coronavirus disease 2019
CPE: Cytopathic effect
CT value: Cycle threshold value
ICU: Intensive care unit
IFA: Immunofluorescence assay
IgG: Immunoglobulin G
NPS: Nasopharyngeal swabs
PCR: Polymerase chain reaction
RNA: Ribonucleic acid
RT-PCR: Real time - polymerase chain reaction
SARS-CoV-2: Severe acute respiratory syndrome coronavirus 2
